# Intrauterine growth restriction alters growth performance, plasma hormones, and small intestinal microbial communities in growing-finishing pigs

**DOI:** 10.1186/s40104-020-00490-x

**Published:** 2020-08-19

**Authors:** Liang Xiong, Jinming You, Wanghong Zhang, Qian Zhu, Francois Blachier, Yulong Yin, Xiangfeng Kong

**Affiliations:** 1 CAS Key Laboratory of Agro-ecological Processes in Subtropical Regions, Hunan Provincial Key Laboratory of Animal Nutritional Physiology and Metabolic Process, National Engineering Laboratory for Pollution Control and Waste Utilization in Livestock and Poultry Production, Institute of Subtropical Agriculture, Chinese Academy of Sciences, Changsha, 410125 Hunan China; 2 Key Laboratory of Animal Nutrition in Jiangxi Province, College of Animal Science and Technology, Jiangxi Agricultural University, Nanchang, 440000 Jiangxi China; 3 Université Paris-Saclay, AgroParisTech, INRAE, UMR PNCA, 75005 Paris, France

**Keywords:** Growing-finishing pigs, Growth performance, Intrauterine growth restriction, Microbial community, Small intestine

## Abstract

**Background:**

The interaction of the gut microbiota with key metabolic and physiological processes may be associated with poor growth outcomes in animals born with intrauterine growth restriction (IUGR).

**Results:**

Growth performance, plasma hormone concentrations, and intestinal microbiota composition were analyzed in IUGR pigs and in normal birth weight (NBW) pigs when the NBW pigs reached 25, 50, and 100 kg of body weight (BW). Compared to NBW pigs, IUGR pigs had lower initial, weaned, and final BW, and lower average daily gain and average daily feed intake in all the considered time points. In the 25 kg BW group, IUGR pigs had higher concentrations of plasma ghrelin and pancreatic polypeptide (PP), but lower insulin concentration than NBW pigs, while the situation was reversed in the 50 kg BW group. As compared to NBW pigs, IUGR pigs had higher microbial alpha diversity in the jejunum and ileum; in the 50 and 100 kg BW groups, IUGR pigs had higher Firmicutes abundance but lower Proteobacteria abundance in the jejunum, and lower *Lactobacillus* abundance in the jejunum and ileum; in the 25 kg BW group, IUGR pigs showed higher unclassified Ruminococcaceae abundance in the ileum; and in 25 and 50 kg BW groups, IUGR pigs showed lower *Ochrobactrum* abundance in the jejunum. Spearman’s correlation revealed that *Lactobacillus* was negatively correlated with growth performance, while unclassified Ruminococcaceae was positively correlated. Predictive metagenomic analysis detected significantly different expression of genes in the intestinal microbiota between IUGR and NBW pigs, suggesting different metabolic capabilities between the two groups.

**Conclusions:**

Growing-finishing IUGR pigs showed lower growth performance, higher microbial alpha diversity, and differences in plasma hormone concentrations compared to NBW pigs. Alterations in the abundance of Firmicutes, Proteobacteria, Ruminococcaceae, *Lactobacillus,* and *Ochrobactrum* in the small intestine may be associated with IUGR, and may therefore serve as a future target for gut microbiota intervention in growing-finishing IUGR pigs.

## Background

Intrauterine growth restriction (IUGR), defined as the impaired growth and development of a mammalian embryo/fetus or fetal organs during pregnancy, is a major concern in pig farming [1]. Animals with IUGR are characterized by feeding intolerance, gut dysfunction, increased susceptibility to infection, and postnatal long-term growth limitation, resulting in higher morbidity and mortality early in their postnatal life [2]. IUGR occurs in 15–20% of newborn piglets and causes considerable economic losses in large-scale pig production farms [3]. Therefore, preventing IUGR and/or correcting its deleterious effects in growing animals is an important goal to improve the economic efficiency of pig production.

The small intestine, in addition to its role in digestion and nutrient absorption, is responsible for most immune system activities in mammals [4]. Previous studies confirmed that IUGR reduces the weight of the small intestine at birth and impairs its functions [5]. From a molecular perspective, IUGR is associated with modifications in the developmental pattern of the intestinal structure as well as with changes in the transcriptomic and proteomic profiles [6], which reduce the intestinal barrier function [7]. Therefore, the delay and alteration of gut development in piglets by IUGR are likely to play a major role in the slower growth rate [8].

The mammalian gastro-intestine harbors a large microbial community, the microbiota [9]. In pigs, the intestinal microbiota is involved in digestion and in the utilization of endogenous or acquired diet carbohydrates and proteins, production of vitamins, maintenance of intestinal morphology, regulation of immune responses, and establishment of the initial (innate) immune defense [10]. In addition, the intestinal microbiota synthesizes a large variety of metabolites, starting from dietary precursors, which are known to positively affect the energy metabolism and functions of the intestinal epithelial cells [11]. In addition, IUGR pigs are characterized by a different plasma concentration patterns for insulin, growth hormone, and insulin-like growth hormone, which can decisively affect the metabolic function, growth, and development of piglets [12, 13].

The existence of a link between intestinal microbiota and growth limitation in IUGR pigs is still unclear. In our previous study, the effects of IUGR on growth performance and intestine microbial community had been reported at 7, 21, and 28 d of age during the lactation [14]. Thus, the present study was conducted to determine the effects of IUGR on gut microbiota of growing-finishing pigs to investigate whether the subsequent performance are the carryover effect from the lactation. In the present study, we measured the differences in growth performance, concentration of plasma hormones, and small intestine microbiota profiles between IUGR and normal birth weight (NBW) pigs, to determine if IUGR is associated with long-lasting alterations in intestinal microbiota composition. A possible causal link between intestinal microbiota composition differences and changes in the physiological parameters is also discussed. The results obtained here could help identify plasma hormone- and intestinal microbiota-related biomarkers associated with growth performance in IUGR animals.

## Materials and methods

### Experimental design, animals, and diets

A total of 36 parity 3 and 4 sows (Large White and Landrace) from three farrowing groups (12 sows each group) were selected from an experimental herd located in Yong’an Town, Liuyang City, Hunan Province, China. After delivery, 72 castrated male piglets were obtained from a total of 36 litters with 10 to 12 born alive piglets, including one IUGR piglet and one NBW piglet per litter. Piglets with a birth weight greater than the mean birth weight per litter were classified as NBW piglets, while piglets with the lowest birth weight per litter were classified as IUGR piglets. Our study selected pregnant sows of three different farrowing groups at different periods and the piglets used in this study were from three farrowing groups for 25, 50, and 100 kg body weight (BW) groups. Thus, this study was divided into three independent trials.

Piglets were weaned at 27 d of age and transferred to a nursery facility. To reduce the influence of mutual attack and psychological stress of weaned piglets after commingling, the pigs were individually penned in this study. The piglets were housed in an environmentally controlled facility with hard plastic and slatted flooring. Each 0.6 m × 1.2 m pen was equipped with a single-hole feeder and a water nipple to allow ad libitum consumption of feed (provided twice daily at 8:00 and 16:00) and drinking water. Therefore, the pigs were remained in nursery facility until they reached 165 d of age. A nursery diet was fed at 28–69 d of age, a growing diet at 70–103 d of age, and a finishing diet at 104–165 d of age (Table 1). The study was completed when average body weight of NBW pigs reached 25, 50, and 100 kg for 25 kg BW, 50 kg BW, and 100 kg BW groups, respectively. Pigs were fed in three phases feeding regime which diets were offered to the pigs as pellets for 25 kg, 50 kg, and 100 kg BW groups, respectively. The change of phases was made when the average BW of NBW pigs for 25 kg, 50 kg, and 100 kg BW groups reached 25 kg, 50 kg, and 100 kg. The dietary nutrient levels were referred to NRC (2012) recommendation and considered the commercial pig production. The diets used in this study were provided by the pig farm. No antibiotics were used during the entire study. The NBW and IUGR piglets from each litter had the same source of breast-milk, farrowing pens, and growth environment during lactation, to ensure that the initial colonization of intestinal microbiota was similar.

**Table 1:** Composition and nutrient levels of experimental diets (as air-dried)

Items	Nursery pig feed (28–69 d of age)	Growing pig feed (70–103 d of age)	Finishing pig feed (104–165 d of age)
Ingredients, %			
Corn	60.00	61.00	61.17
Barley	6.00	8.00	8.00
Soybean oil	2.00	1.50	1.00
Soybean meal	27.50	25.00	25.50
CaHPO_4_	0.10	0.10	0
Lysine	0.16	0.18	0.13
Methionine	0.02	0.03	0.00
Threonine	0.10	0.07	0.08
Anti-oxidant	0.02	0.02	0.02
Anti-mildew agent	0.10	0.10	0.10
Nursery pigs premix^a^	4.00	0	0
Growing-finishing pigs premix^b^	0	4.00	4.00
Total	100.00	100.00	100.00
Nutrient levels^c^			
Digestible energy, MJ/kg	13.91	13.77	13.64
Crude protein	17.20	16.40	16.50
Crude fat	4.70	4.30	3.80
Crude fiber	2.70	2.70	2.80
Digestible lysine	1.17	1.08	1.05
Digestible methionine	0.33	0.30	0.28
Digestible threonine	0.77	0.71	0.73
Total calcium	0.77	0.74	0.66
Total phosphorus	0.56	0.52	0.45

^a^Nursery pig premix supplied per kg feed: 8,000 IU vitamin A, 228 IU vitamin D_3_, 15 IU vitamin E, 3.0 mg vitamin K_3_, 1.3 mg vitamin B_1_, 3.1 mg vitamin B_2_, 1.2 mg vitamin B_6_, 0.03 mg vitamin B_12_, 13.4 mg calcium pantothenate, 500 mg choline chloride, 120 mg iron, 10 mg copper, 130 mg zinc, 100 mg manganese, 0.3 mg iodine, and 0.3 mg selenium

^b^Growing-finishing pig premix supplied per kg feed: 15,000 IU vitamin A, 200 IU vitamin D_3_, 50 IU vitamin E, 4.0 mg vitamin K_3_, 4.0 mg vitamin B_1_, 10 mg vitamin B_2_, 3.0 mg vitamin B_6_, 0.04 mg vitamin B_12_, 20.0 mg calcium pantothenate, 800 mg choline chloride, 120 mg iron, 20 mg copper, 112 mg zinc, 124 mg manganese, 0.5 mg iodine, 0.4 mg selenium

^c^Nutrient levels were calculated values

### Growth performance

Each pig was weighed at birth (initial BW), at weaning (27 days old) and at the end of each stage (including nursery, growing, and finishing), to calculate average daily gain (ADG). Feed intake and remaining feed per pen were recorded weekly and at the end of each stage to calculate the average daily feed intake (ADFI) and feed/gain (F/G) ratio.

### Sample collection

When the average BW of NBW pigs reached 25, 50, and 100 kg BW, 10 pigs per group were randomly weighed and blood samples were collected from the precaval vein 12 h after the last feeding, for plasma hormone concentration determination. The sampled animals were exsanguinated after electrical stunning. The luminal contents of the jejunum (10 cm below the flexura of duodenum-jejunum) and ileum (10 cm above the ileocecal junction) were sampled and stored at −80 °C for microbiota composition analysis.

### Concentration of plasma hormones

Plasma concentrations of gastrin, growth hormone (GH), ghrelin, glucagon, insulin-like growth factor-1 (IGF-1), insulin, leptin, pancreatic polypeptide (PP), peptide YY (PYY), and somatostatin (SS) were assayed using the Meimian ELISA kit (Suzhou Yutong Biotechnology Company, Suzhou, Jiangsu, China), as per the manufacturer’s instructions, and then read on a spectrophotometer (Biomate 5, Thermo Electron Corporation, Rochester, NY, USA).

### Microbiota DNA isolation and PCR amplification

Total bacterial genomic DNA was extracted from intestinal samples using the Fast DNA SPIN extraction kit (MP Biomedicals, Santa Ana, CA, USA) following the manufacturer’s instructions, and stored at −20 °C until further analysis. The quantity and quality of the extracted DNA were assessed using a NanoDrop ND-1000 spectrophotometer (Thermo Fisher Scientific, Waltham, MA, USA) and agarose gel electrophoresis, respectively.

The bacterial 16S rRNA gene V3–V4 region was amplified by PCR using the forward primer 338F (5′-ACTCCTACGGGAGGCAGCA-3′) and the reverse primer 806R (5′-GGACTACHVGGGTWTCTAAT-3′). Sample-specific 7-bp barcodes were added to the primers for multiplex sequencing. The PCR mix included the following components: 5 μL Q5 reaction buffer (5×), 5 μL Q5 High-Fidelity GC buffer (5×), 0.25 μL Q5 High-Fidelity DNA Polymerase (5 U/μL), 2 μL (2.5 mmol/L) dNTPs, 1 μL each (10 μmol/L) of forward and reverse primers, 2 μL DNA template, and 8.75 μL double distilled water. The Quant-iT™ PicoGreen™ dsDNA Assay Kit used in the PCR assay was purchased from Thermo Fisher Scientific (USA). Amplification reactions were carried out with the following profile: 2 min of initial denaturation at 98 °C, 25 cycles of 15 s at 98 °C, 30 s at 55 °C, and 30 s at 72 °C, and final extension at 72 °C for 5 min. Amplicons were further purified using Agencourt AMPure Beads (Beckman Coulter, Indianapolis, IN, USA) and quantified using the PicoGreen dsDNA assay kit (Invitrogen, Carlsbad, CA, USA) following the manufacturer’s protocols. Purified amplicons were grouped in equimolar pools, and paired-end (2 × 300 bp) sequencing was performed using the MiSeq Reagent Kit v3 (600 cycles) on an Illumina MiSeq platform (Illumina, San Diego, CA, USA), according to the standard protocols established by Shanghai Personal Biotechnology Co. Ltd. (Shanghai, China).

### Statistical analyses

Significance of the differences in growth performance and plasma hormone profile between IUGR and NBW pigs was assessed by the Student’s *t*-test and for microbiota alpha diversity and abundance by the Mann-Whitney U-test. Both analyses were performed on SPSS 22.0 (Chicago, IL, USA). Alpha diversity (ACE, Chao1, Shannon, and Simpson indices) was assessed in QIIME 1.8 (http://qiime.org/). The beta diversity was assessed to investigate structural variation in the intestinal microbiota among samples by principal coordinate analysis (PCoA) based on unweighted UniFrac distance. Partial least squares discriminant analysis (PLS-DA) based on unweighted UniFrac distance with constrained ordination and supervised learning was also performed to reveal the intestinal microbiota variation among samples [15]. Linear discriminant analysis (LDA) effect size (LEfSe) and statistical analysis of metagenomic profiles (STAMP) 2.1.3 software were used to simultaneously compare differences in taxonomic levels, including phylum and genus, between IUGR and NBW pigs. Phylogenetic Investigation of Communities by Reconstruction of Unobserved States (PICRUSt) was used to characterize the functional capacity of the small intestine microbiota of growing-finishing pigs. In LEfSe analysis, the non-parametric factorial Kruskal–Wallis (KW) sum-rank test was used to detect all species with significant differential abundance and the Wilcoxon rank-sum test was used to investigate biological consistency among subclasses [16]. As a last step, histograms of the LDA score were used to measure the effect size for determining the significantly different taxa and metabolic functions based on the observed relative differences. Spearman’s correlation between growth performance and intestinal microbiota composition was calculated using the R package ggplot2 3.3.1 (https://www.r-project.org/). GraphPad Prism 6.0 (San Diego, CA, USA) was used to plot the images. Data are shown as means ± SEM. Differences between IUGR pigs and NBW pigs were considered significant when the *P*-value < 0.05.

## Results

### Growth performance

In the 25 and 50 kg BW groups, IUGR pigs had significantly lower initial BW, weaned BW, final BW, ADG, and ADFI (*P* < 0.05) than NBW pigs, but we observed no significant differences in F/G (Table 2). In the 100 kg BW group, IUGR pigs had significantly lower initial BW, weaned BW, final BW, and ADG than NBW pigs (*P* < 0.05), but ADFI and F/G were not significantly different.

**Table 2:** Effect of intrauterine growth restriction (IUGR) on growth performance of growing-finishing pigs

Items	25 kg BW group		50 kg BW group		100 kg BW group	
	NBW	IUGR	NBW	IUGR	NBW	IUGR
Initial BW, kg	1.77 ± 0.09	1.04 ± 0.07^**^	1.79 ± 0.06	1.01 ± 0.03^**^	1.86 ± 0.10	0.96 ± 0.04^**^
Weaned BW, kg	10.39 ± 0.37	7.23 ± 0.51^**^	13.03 ± 0.47	8.59 ± 0.66^**^	6.57 ± 0.34	3.66 ± 0.23^**^
Final BW, kg	26.33 ± 0.81	17.84 ± 0.89^*^	46.86 ± 3.25	31.42 ± 2.55^**^	105.40 ± 3.51	81.71 ± 3.23^**^
ADG, kg/d	0.59 ± 0.01	0.43 ± 0.01^*^	0.65 ± 0.15	0.44 ± 0.08^*^	0.69 ± 0.08	0.55 ± 0.07^**^
ADFI, kg/d	0.96 ± 0.04	0.72 ± 0.03^**^	1.48 ± 0.34	0.92 ± 0.12^*^	1.92 ± 0.40	1.48 ± 0.26
F/G	1.64 ± 0.04	1.67 ± 0.08	2.28 ± 0.36	2.09 ± 0.16	2.78 ± 0.13	2.71 ± 0.22

Data are presented as means ± SEM. The data presented are obtained from 12 animals in each group (*n* = 12). In the same row, values with * were significantly different from NBW pigs at *P* < 0.05 and values with ** were significantly different at *P* < 0.01. *ADG*, average daily gain; *ADFI*, average daily feed intake; *BW*, body weight; *F/G*, feed/gain; *Initial BW*, body weight at birth; *NBW*, normal born weight

### Plasma hormone profile

Compared to NBW pigs, IUGR pigs in the 25 kg BW group had higher concentrations of plasma ghrelin, SS, PYY, and PP, but lower concentration of insulin (*P* < 0.05). The IUGR pigs in the 50 kg BW group had higher concentrations of IGF-1, leptin, and insulin, but lower concentrations of gastrin, ghrelin, and PP (*P* < 0.05) than NBW pigs. No significant differences were observed between IUGR and NBW pigs in the 100 kg BW group (Table 3).

**Table 3:** Effect of intrauterine growth restriction (IUGR) on plasma hormones of growing-finishing pigs

Items	25 kg BW group		50 kg BW group		100 kg BW group	
	NBW	IUGR	NBW	IUGR	NBW	IUGR
Gastrin, ng/mL	0.48 ± 0.03	0.50 ± 0.02	2.87 ± 0.53	1.68 ± 0.18*	1.32 ± 0.15	1.49 ± 0.09
GH, ng/mL	17.50 ± 0.58	18.27 ± 0.63	47.38 ± 12.07	32.24 ± 4.43	29.13 ± 2.68	30.05 ± 2.05
Ghrelin, ng/mL	4.81 ± 0.23	5.81 ± 0.26*	21.34 ± 4.30	11.19 ± 1.30*	9.40 ± 1.24	10.62 ± 0.67
Glucagon, ng/mL	0.32 ± 0.01	0.33 ± 0.05	0.52 ± 0.13	0.59 ± 0.08	0.40 ± 0.04	0.43 ± 0.02
IGF-1, ng/mL	125.65 ± 5.09	140.27 ± 6.12	255.18 ± 21.76	399.38 ± 60.88*	284.78 ± 30.20	269.82 ± 12.57
Insulin, mIU/L	33.27 ± 0.93	29.72 ± 1.14*	37.65 ± 2.02	46.61 ± 3.42*	38.02 ± 4.64	37.52 ± 4.15
Leptin, ng/mL	10.69 ± 0.28	12.08 ± 0.74	17.88 ± 0.98	29.38 ± 4.94*	24.93 ± 3.04	22.99 ± 1.82
PP, ng/mL	2.09 ± 0.07	2.65 ± 0.10**	12.01 ± 1.50	6.81 ± 0.98**	6.82 ± 0.94	7.69 ± 0.70
PYY, pmol/mL	9.98 ± 0.39	14.91 ± 0.61**	28.54 ± 6.65	24.26 ± 4.98	19.83 ± 2.08	18.95 ± 1.09
SS, pg/mL	89.11 ± 3.17	98.44 ± 1.24*	193.49 ± 59.58	180.15 ± 36.94	145.80 ± 15.03	133.19 ± 10.92

Data are presented as means ± SEM. The data presented are obtained from 10 animals in each group (*n* = 10). In the same row, values with * were significantly different from NBW pigs at *P* < 0.05 and values with ** were significantly different at *P* < 0.01. *GH* Growth hormone; *IGF-1* Insulin-like growth factors-1, *NBW* Normal born weight, *PP* Pancreatic polypeptide, *PYY* Peptide YY, *SS* Somatostatin

### 16S rRNA sequencing data

A total of 4,237,106 high-quality DNA sequences were obtained from the high-throughput sequencing of 120 samples, which included 60 jejunum and 60 ileum samples from growing-finishing pigs in the 25, 50, and 100 kg BW groups. We randomly analyzed a subsample of 27,223 sequences for each sample, to avoid bias caused by different sequencing depths. The rarefaction curves obtained suggested that almost all bacterial species were captured using this approach (Supplementary Fig. 1).

### Microbiota diversity in the small intestine

**Fig. 1:**
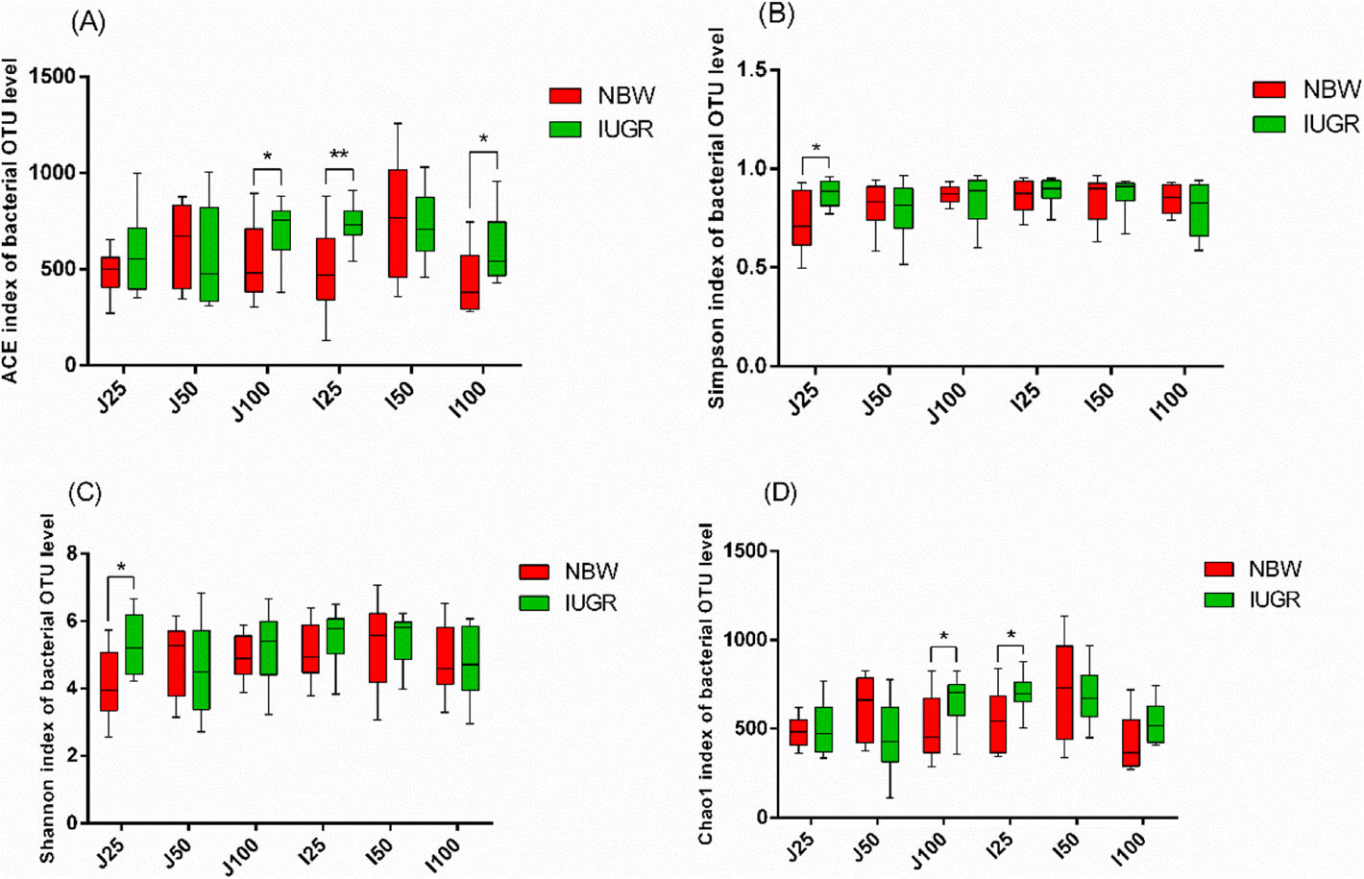
Microbial alpha diversity in the jejunum and ileum contents of intrauterine growth retardation (IUGR) pigs and normal birth weight (NBW) pigs in the 25, 50, and 100 kg BW groups. The data presented are obtained from 10 animals in each group (*n* = 10). **a** ACE index, **b** Simpson index, **c** Shannon index, and **d** Chao1 index. * denotes a significant difference (*P* < 0.05) among different treatments

Differences in alpha diversity, including ACE (Fig. 1a), Simpson (Fig. 1b), Shannon (Fig. 1c), and Chao1 (Fig. 1d) indices, were assessed by performing the Mann–Whitney U-test. In the jejunum, IUGR pigs in the 25 kg BW group had significantly higher values of Simpson and Shannon indices than NBW pigs (*P* < 0.05); in the 100 kg BW group, ACE and Chao1 indices were also significantly higher in IUGR pigs. In the ileum, IUGR pigs in the 25 kg BW group had higher ACE (*P* < 0.01) and Chao1 (*P* < 0.05) indices than NBW pigs, but those in the 100 kg BW group had lower ACE (*P* < 0.05).

**Fig. 2:**
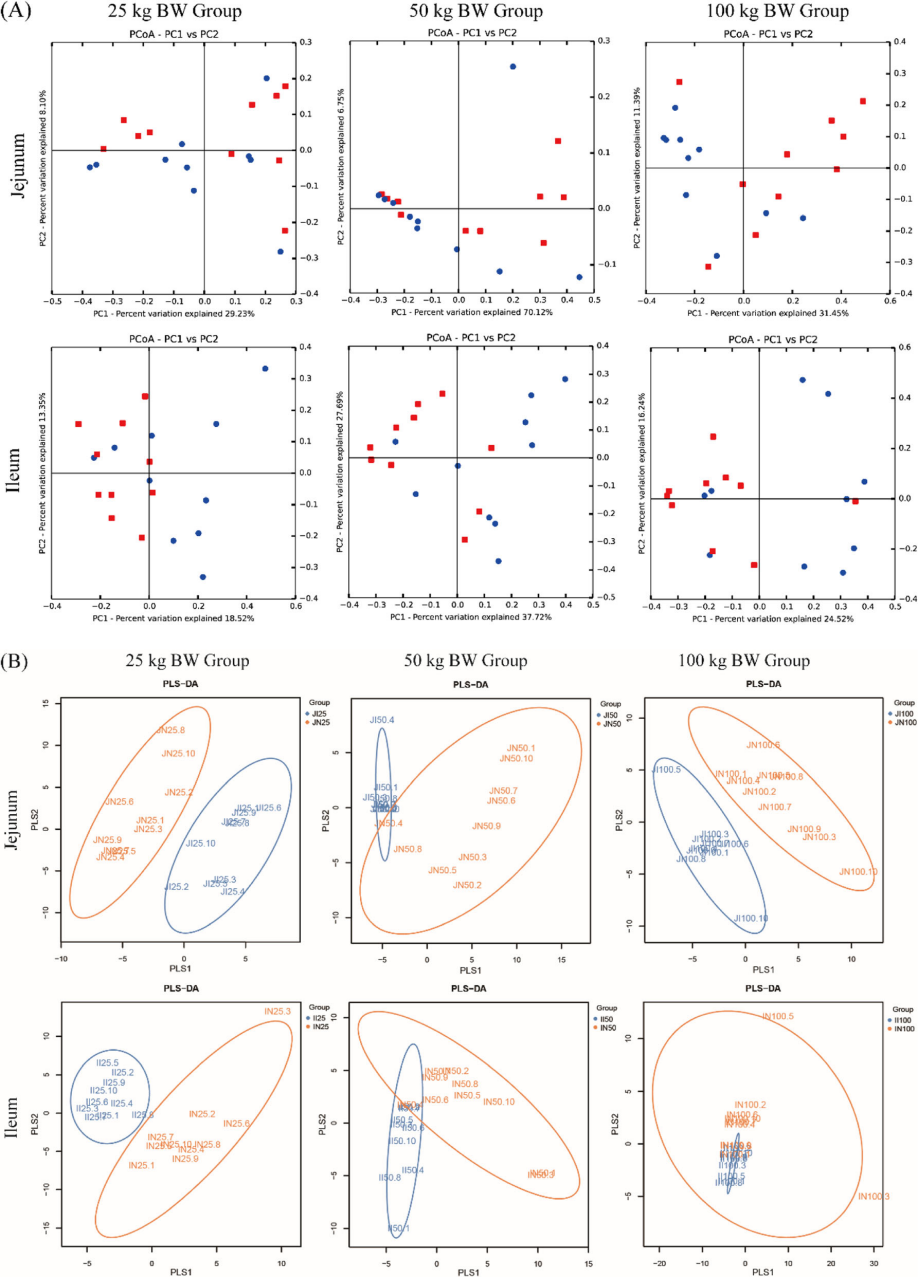
Differences in intestinal microbial community structure between intrauterine growth retardation (  IUGR) pigs and normal birth weight (  NBW) pigs in the 25, 50, and 100 kg BW groups, and each symbol represents the intestinal microbiota of one pig. The data presented are obtained from 10 animals in each group (*n* = 10). **a** Principal coordinate analysis (PCoA) along the axes IUGR and NBW. **b** Partial least square discriminant analysis (PLS-DA) based on an unweighted UniFrac distances score plot of jejunum and ileum microbiota. Each point represents the microbial community of one pig

The beta-diversity analysis was performed to measure the dissimilarity of microbial communities between IUGR pigs and NBW pigs. PCoA plots did not indicate a clear separation between NBW pigs and IUGR pigs (Fig. 2). We thus performed a PLS-DA based on unweighted UniFrac distances. This approach revealed that microbial communities in the jejunum of NBW and IUGR pigs in the 25, 50, and 100 kg BW groups and in the ileum of NBW and IUGR pigs in the 25 and 50 kg BW groups were clearly separated and clustered into distinct groups.

### Microbial community composition in the small intestine

**Fig. 3:**
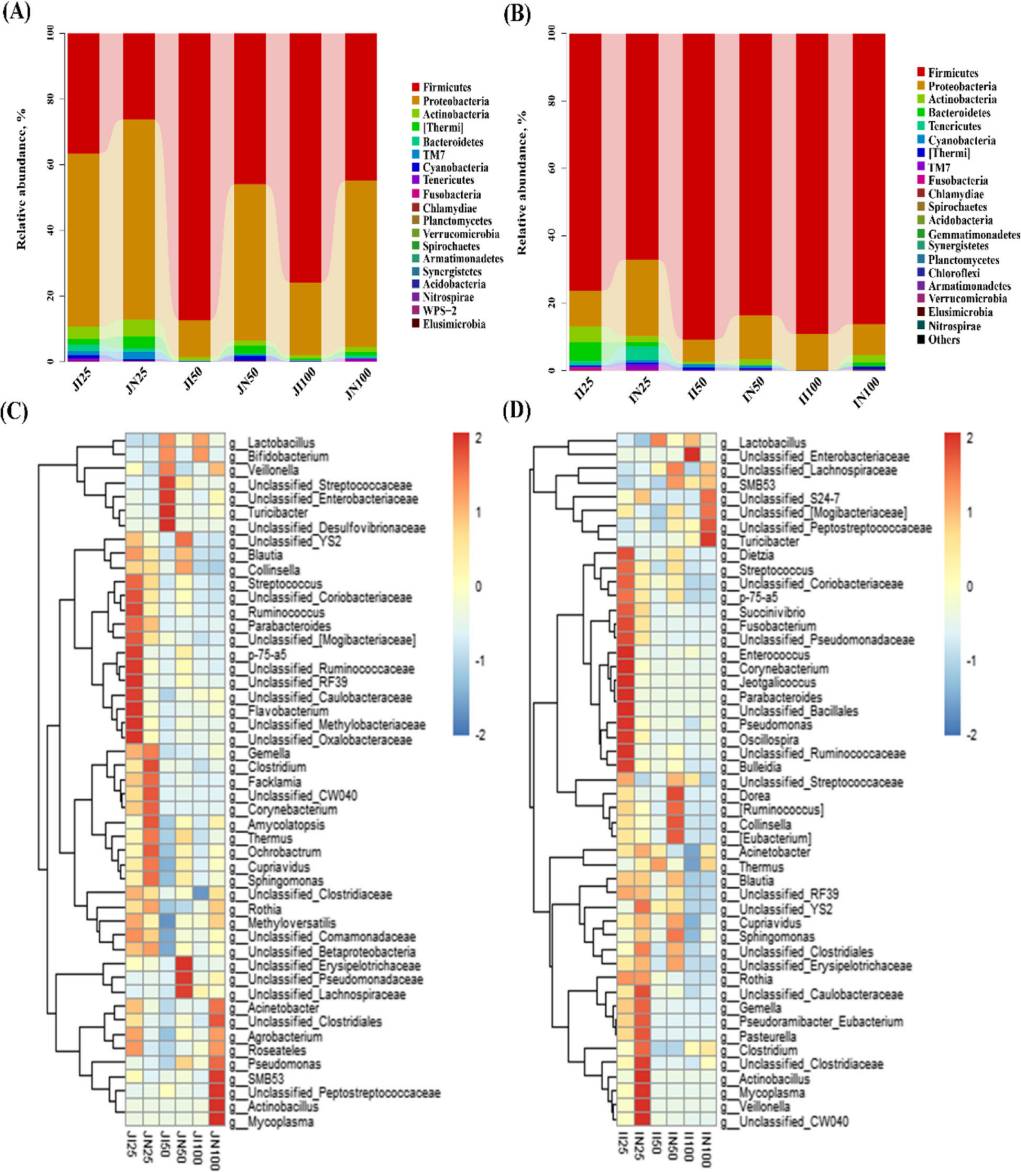
Phylum-level microbial community structure in the jejunum (**a**) and ileum (**b**) and genus-level structure in the jejunum (**c**) and ileum (**d**) in intrauterine growth retardation (IUGR) pigs and normal birth weight (NBW) pigs. The data presented are obtained from 10 animals in each group (*n* = 10). Relative abundances of microbial phyla with > 0.01% proportion and genera for the top 50 relative abundance are listed. JI and JN represent samples obtained from the jejunum of IUGR pigs and NBW pigs, respectively; II and IN represent samples obtained from the ileum of IUGR pigs and NBW pigs, respectively. Twenty-five, 50, and 100 represent 25, 50, and 100 kg BW groups

Based on 97% 16S rRNA gene sequence identity, we identified 19 microbial phyla and 221 microbial genera in the jejunum and 21 phyla and 223 genera in the ileum of IUGR and NBW pigs (Fig. 3).

Microbiota composition analysis showed that Firmicutes, Proteobacteria, Actinobacteria, and Bacteroidetes were the dominant microbial phyla in both IUGR pigs and NBW pigs in all BW groups. In the jejunum (Fig. 3a), Firmicutes, Proteobacteria, Actinobacteria, and Thermi were the dominant microbial phyla in NBW pigs of all BW groups, while Firmicutes, Proteobacteria, Actinobacteria, and Bacteroidetes were the most abundant in IUGR pigs. In the ileum (Fig. 3b), Firmicutes and Proteobacteria were the most abundant phyla, followed by Actinobacteria and Bacteroidetes in both IUGR pigs and NBW pigs. Other microbial phyla were present at very low relative abundances.

The abundance distribution in intestinal microbiota at the genus level was plotted as a heatmap (Fig. 3c). For IUGR pigs in the 25 kg BW group, the four dominant genera in the jejunum were *Cupriavidus* (29.18%), *Streptococcus* (12.87%)*, Acinetobacter* (6.14%), and unclassified Clostridiaceae (4.66%). In the 50 kg BW group, the most abundant genera were *Lactobacillus* (73.63%), unclassified Desulfovibrionaceae (6.87%)*, Turicibacter* (3.79%), and unclassified Enterobacteriaceae (3.01%). In the 100 kg BW group, *Lactobacillus* (68.46%), *Cupriavidus* (12.80%), *Streptococcus* (3.19%), and *Pseudomonas* (2.44%) were the most abundant genera. For NBW pigs, the dominant genera in the jejunum were *Cupriavidus* (46.35%), *Streptococcus* (9.09%)*, Acinetobacter* (6.14%), and unclassified Caulobacteraceae (3.91%) in the 25 kg BW group; *Cupriavidus* (28.57%), *Lactobacillus* (26.21%), *Streptococcus* (6.57%), and *Pseudomonas* (5.25%) in the 50 kg BW group; and *Cupriavidus* (22.54%), *Lactobacillus* (20.64%), *Acinetobacter* (8.15%), and *Pseudomonas* (7.79%) in the 100 kg BW group.

For IUGR pigs, the dominant genera in the ileum were *Streptococcus* (28.23%), *Lactobacillus* (16.12%)*,* unclassified Clostridiaceae (12.03%), and unclassified Ruminococcaceae (7.11%) in the 25 kg BW group; *Lactobacillus* (77.58%), unclassified Clostridiaceae (4.65%), *Cupriavidus* (4.31%), and *Streptococcus* (3.08%) in the 50 kg BW group; and *Lactobacillus* (63.94%), unclassified Enterobacteriaceae (9.51%), unclassified Peptostreptococcaceae (9.40%), and *Turicibacter* (4.69%) in the 100 kg BW group (Fig. 3d). For NBW pigs, the dominant genera in the ileum were unclassified Clostridiaceae (28.02%), *Streptococcus* (15.09%)*, Cupriavidus* (9.11%), and *Actinobacillus* (7.97%) in the 25 kg BW group; *Lactobacillus* (35.15%), *Streptococcus* (18.07%), unclassified Peptostreptococcaceae (10.95%), and *Cupriavidus* (9.75%) in the 50 kg BW group; and *Lactobacillus* (26.42%), unclassified Peptostreptococcaceae (22.27%), *Turicibacter* (15.29%), and unclassified Clostridiaceae (10.33%) in the 100 kg BW group.

### Differences in the intestinal microbial communities of IUGR and NBW pigs

**Fig. 4:**
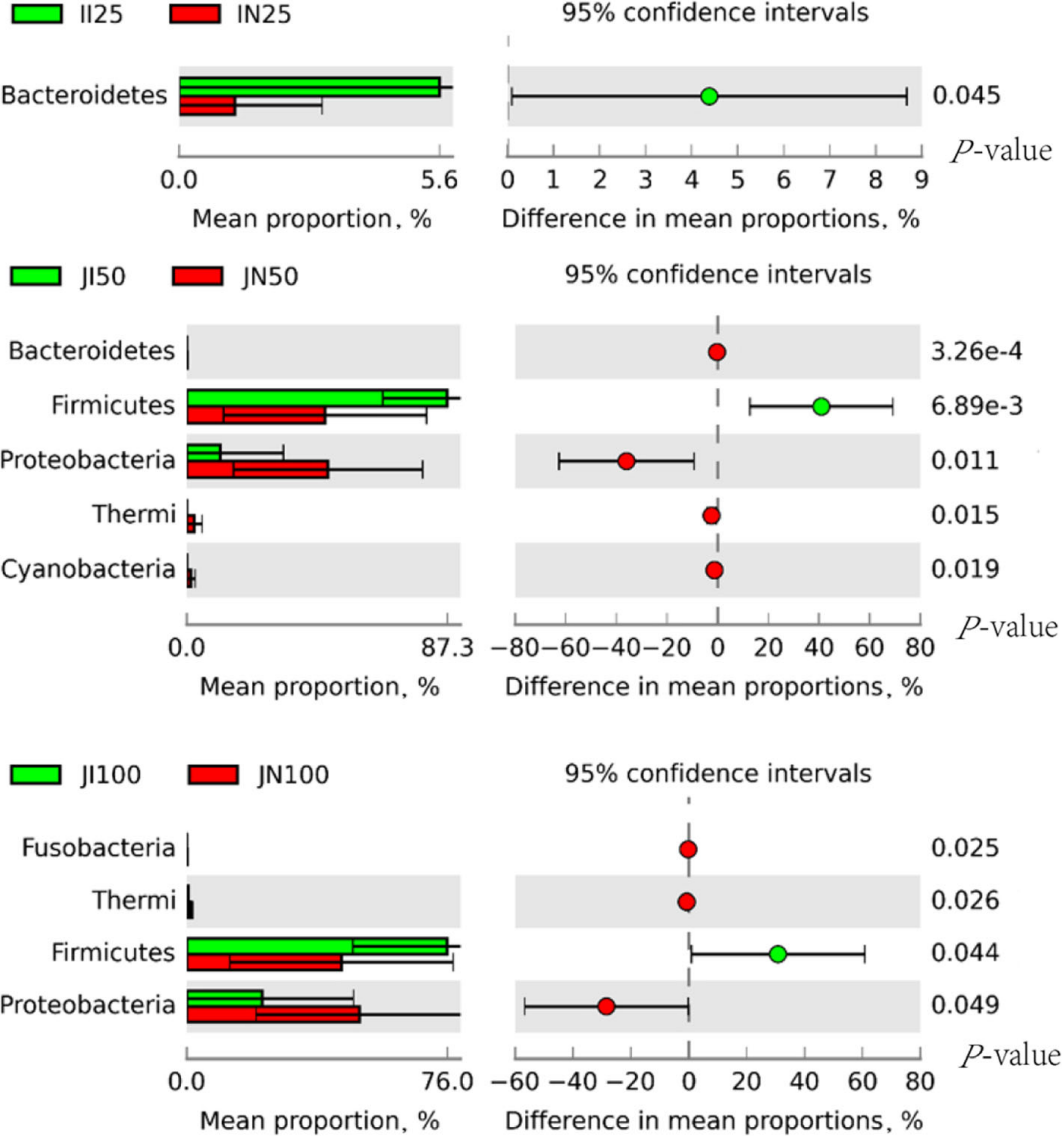
Data for the small intestinal microbial compositions of intrauterine growth retardation (IUGR) pigs and normal birth weight (NBW) pigs at the phylum level were imported into the statistical analysis of metagenomic profiles (STAMP) software for statistical analysis. The data presented are obtained from 10 animals in each group (*n* = 10). Differences were considered significant at *P* < 0.05 using the Mann-Whitney U-test. JI and JN represent samples obtained from the jejunum of IUGR pigs and NBW pigs, respectively; II and IN represent samples obtained from the ileum of IUGR pigs and NBW pigs, respectively. Twenty-five, 50, and 100 represent 25, 50, and 100 kg BW groups

Differences in the relative abundances of the community components of microbiota in the jejunum and ileum of NBW and IUGR pigs were further analyzed using the Mann–Whitney U-test by STAMP and LEfSe. Microbial phyla with relative abundance above 0.01% in the jejunum and in the ileum were selected for analysis (Fig. 4). Compared to NBW pigs, IUGR pigs in the 25 kg BW group had a higher abundance of Bacteroidetes in the ileum (*P* < 0.05), and those in the 50 kg BW group had higher abundance of Firmicutes (*P* < 0.05) and lower abundances of Proteobacteria, Thermi, Cyanobacteria, and Bacteroidetes in the jejunum (*P* < 0.05). In the 100 kg BW group, IUGR pigs had a higher abundance of Firmicutes (*P* < 0.05) and lower abundances of Fusobacteria, Proteobacteria, and Thermi in the jejunum (*P* < 0.05), as compared to NBW pigs. We observed no significant differences in the relative abundance of microbial phyla in the jejunum of pigs in the 25 kg BW group and in the ileum of pigs in the 50 and 100 kg BW groups.

**Fig. 5:**
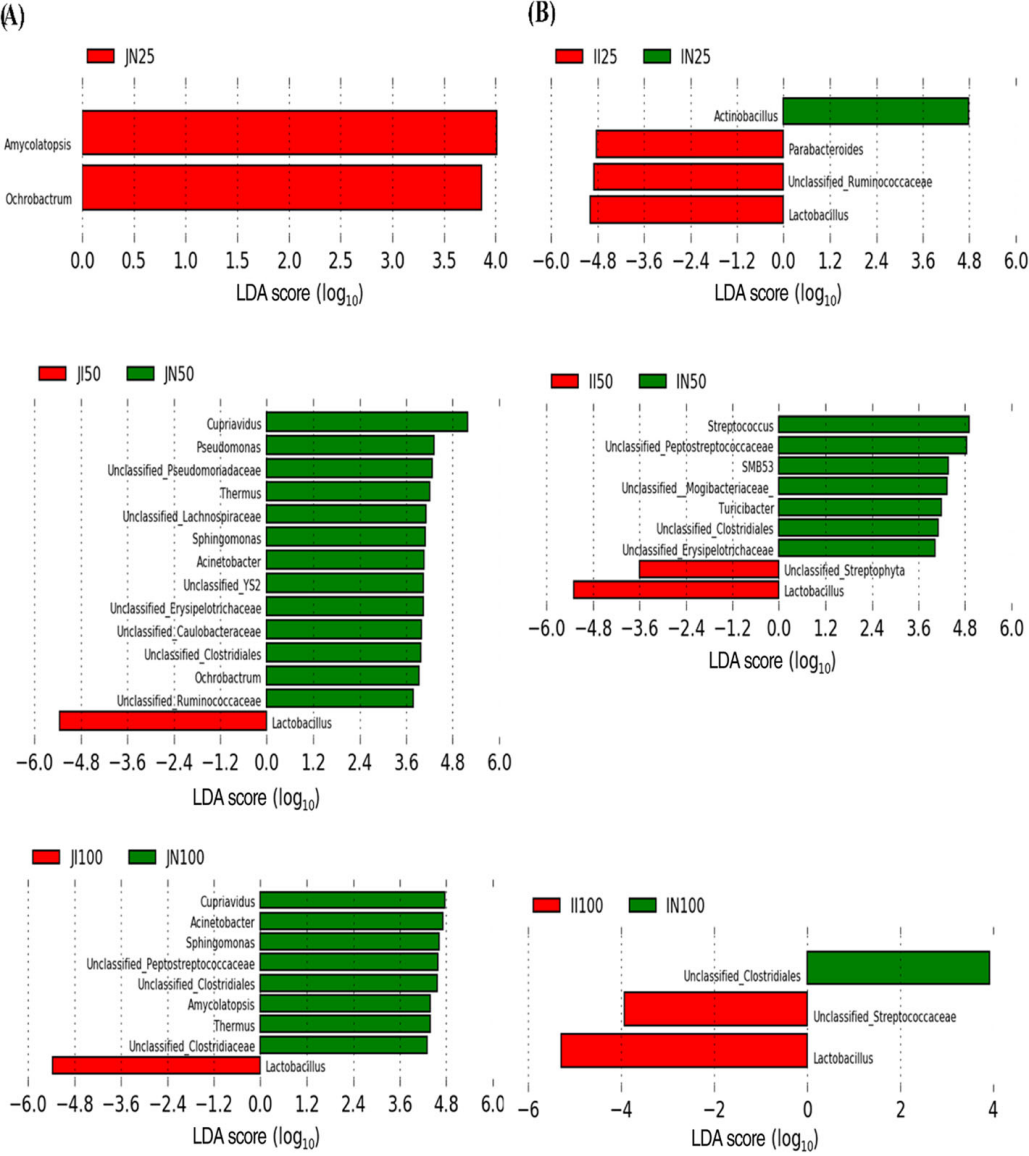
LEfSe analysis of small intestinal microbial compositions of intrauterine growth retardation (IUGR) pigs and normal birth weight (NBW) pigs throughout the trial at genus level. The data presented are obtained from 10 animals in each group (*n* = 10). Significant differences (LDA score ≥ 3, *P* < 0.05) for jejunum and ileum in growing-finishing pigs are shown. JI and JN represent samples obtained from the jejunum of IUGR pigs and NBW pigs, respectively; II and IN represent samples obtained from the ileum of IUGR pigs and NBW pigs, respectively. Twenty-five, 50, and 100 represent 25, 50, and 100 kg BW groups

At the genus level, the top 20 most abundant taxa in the combined samples were analyzed using LEfSe to identify differences, defined as an LDA score above 3.0 (Fig. 5). In the 25 kg BW group, *Amycolatopsis* and *Ochrobactrum* were significantly less abundant in the jejunum of IUGR pigs than in the jejunum of NBW pigs (*P* < 0.05). In the 50 kg BW group, IUGR pigs had significantly higher abundance of *Lactobacillus* in the jejunum but significantly lower abundances of unclassified Ruminococcaceae*, Cupriavidus,* unclassified Pseudomonadaceae*, Pseudomonas*, unclassified Lachnospiraceae, *Ochrobactrum,* unclassified Erysipelotrichaceae, *Sphingomonas*, unclassified Caulobacteraceae, *Acinetobacter*, *Thermus*, unclassified Clostridiales, and unclassified YS2 than NBW pigs (*P* < 0.05). In the 100 kg BW group, IUGR pigs had significantly higher abundance of *Lactobacillus* in the jejunum but significantly lower abundances of *Cupriavidus*, *Thermus*, *Acinetobacter*, *Sphingomonas*, unclassified Clostridiales, *Amycolatopsis*, unclassified Clostridiaceae, and unclassified Peptostreptococcaceae than NBW pigs (*P* < 0.05). In the 25 kg BW group, the abundances of *Lactobacillus,* unclassified Ruminococcaceae, and *Parabacteroides* in the ileum of IUGR pigs was significantly higher (*P* < 0.05), while that of *Actinobacillus* was significantly lower, than in NBW pigs (*P* < 0.05). In the 50 kg BW group, *Lactobacillus* and unclassified Streptophyta had significantly higher abundance in the ileum of IUGR pigs (*P* < 0.05), while *Streptococcus*, unclassified Peptostreptococcaceae, *SMB53*, unclassified Mogibacteriaceae, *Turicibacter,* unclassified Clostridiales, and unclassified Erysipelotrichaceae had significantly lower abundance than in NBW pigs (*P* < 0.05). In the 100 kg BW group, *Lactobacillus* and unclassified Streptococcaceae had higher abundance in the ileum of IUGR pigs, while unclassified Clostridiales had lower abundance, than in NBW pigs (*P* < 0.05).

### Correlation between intestinal microbial community composition and growth performance of pigs

**Fig. 6:**
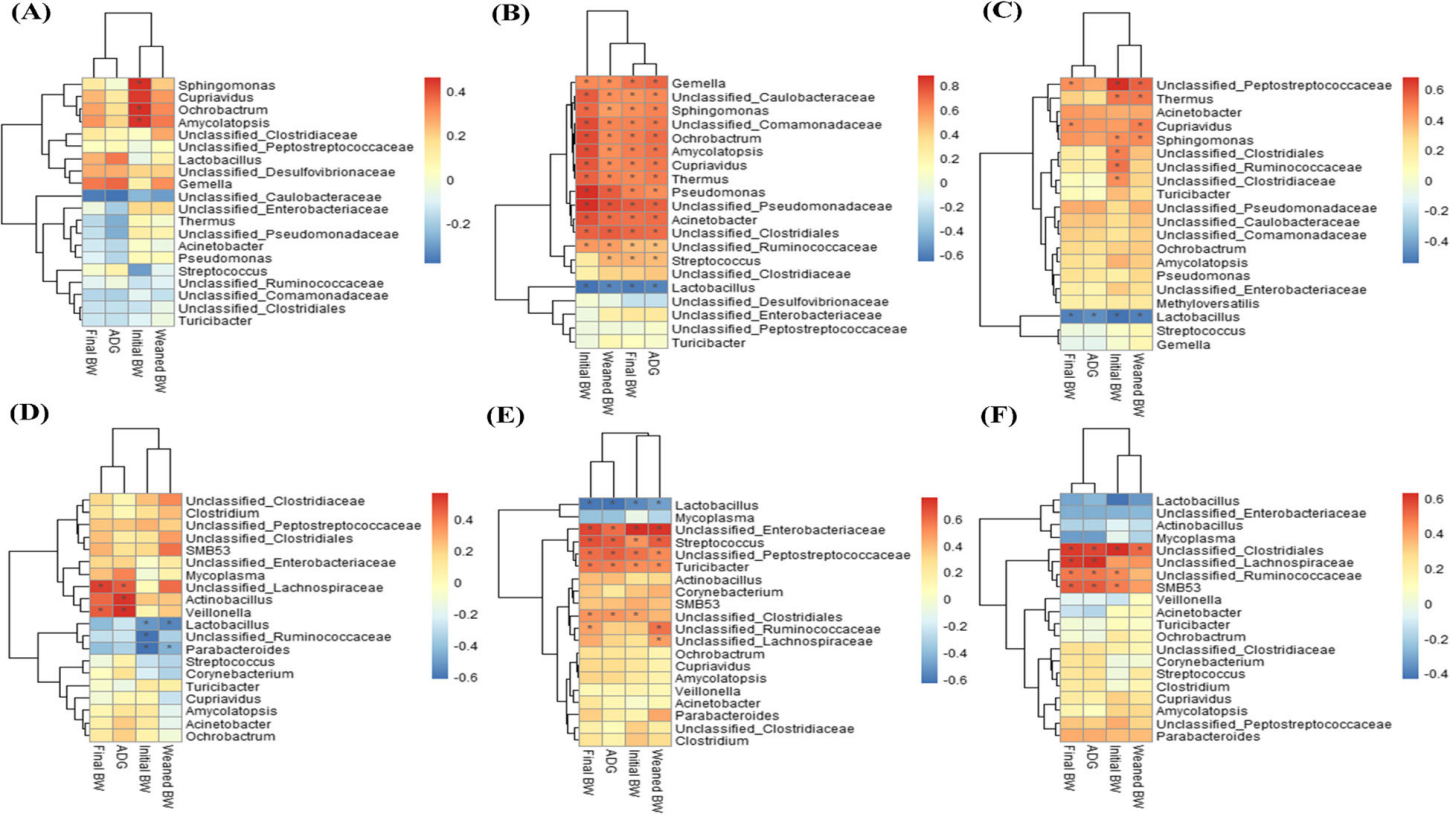
Correlations between the growth performance and most common 20 genera according to relative abundance in the 25 (**a**), 50 (**b**), and 100 (**c**) kg BW groups in the jejunum; and in the 25 (**d**), 50 (**e**), and 100 (**f**) kg BW groups in the ileum. The data presented are obtained from 10 animals in each group (*n* = 10). Average daily gain (ADG), initial body weight (BW at birth), weaned BW, and final BW are presented. Red, blue, and white represent significantly positive correlation, negative correlation, and no significant correlation, respectively. * indicates *P* < 0.05

To analyze the relationship between intestinal microbiota composition and growth performance of growing-finishing pigs, Spearman’s correlations were calculated between the microbial relative abundance of the jejunum and ileum (based on the top 20 abundant taxa at the genus level) and ADG, initial BW, weaned BW, and final BW of IUGR pigs and NBW pigs in the 25, 50, and 100 kg BW groups (Fig. 6).

In the jejunum, *Lactobacillus* was negatively correlated with growth performance. However, unclassified Peptostreptococcaceae, *Thermus*, *Cupriavidus*, *Sphingomonas*, unclassified Clostridiales, unclassified Ruminococcaceae, and unclassified Clostridiaceae were positively correlated with the initial and weaned BW in the 50 (Fig. 6b) and 100 kg BW (Fig. 6c) groups; *Ochrobactrum* and *Amycolatopsis* were positively correlated with the initial BW in the 25 (Fig. 6a) and 50 kg BW groups; and *Gemella*, unclassified Caulobacteraceae, unclassified Comamonadaceae, *Pseudomonas*, unclassified Pseudomonadaceae, *Acinetobacter*, and *Streptococcus* were positively correlated with the growth performance in the 50 kg BW group.

In the ileum, *Lactobacillus* was negatively correlated with initial BW as well as weaned BW in the 25 kg BW (Fig. 6d) group and with growth performance only in the 50 kg BW (Fig. 6e) group. On the contrary, unclassified Ruminococcaceae and unclassified Clostridiales were positively correlated with the growth performance in the 50 and 100 kg BW (Fig. 6f) groups; unclassified Lachnospiraceae was positively correlated with the ADG and final BW in the 25 kg BW group; and unclassified Enterobacteriaceae, unclassified Peptostreptococcaceae, and *Streptococcus* were positively correlated with the growth performance in the 50 kg BW group.

### Metabolic capability profiles of small intestine microbiota

**Fig. 7:**
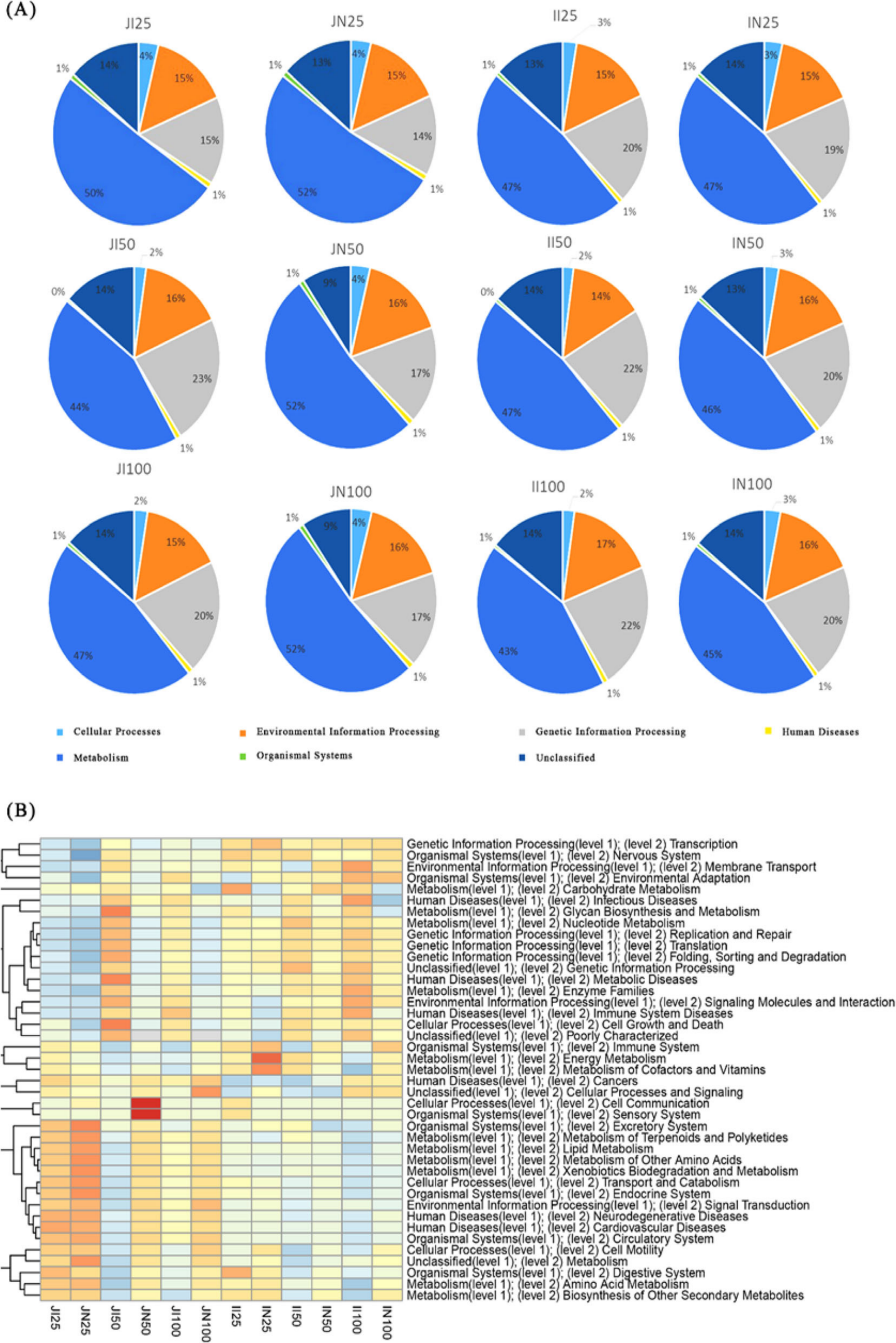
Predictive metagenomics showing differences in function between intrauterine growth retardation (IUGR) pigs and normal birth weight (NBW) pigs in the jejunum and ileum using PICRUSt analysis at level 1 (**a**) and level 2 (**b**). The data presented are obtained from 10 animals in each group (*n* = 10). JI and JN represent samples obtained from the jejunum of IUGR pigs and NBW pigs, respectively; II and IN represent samples obtained from the ileum of IUGR pigs and NBW pigs, respectively. Twenty-five, 50, and 100 represent 25, 50, and 100 kg BW groups

**Fig. 8:**
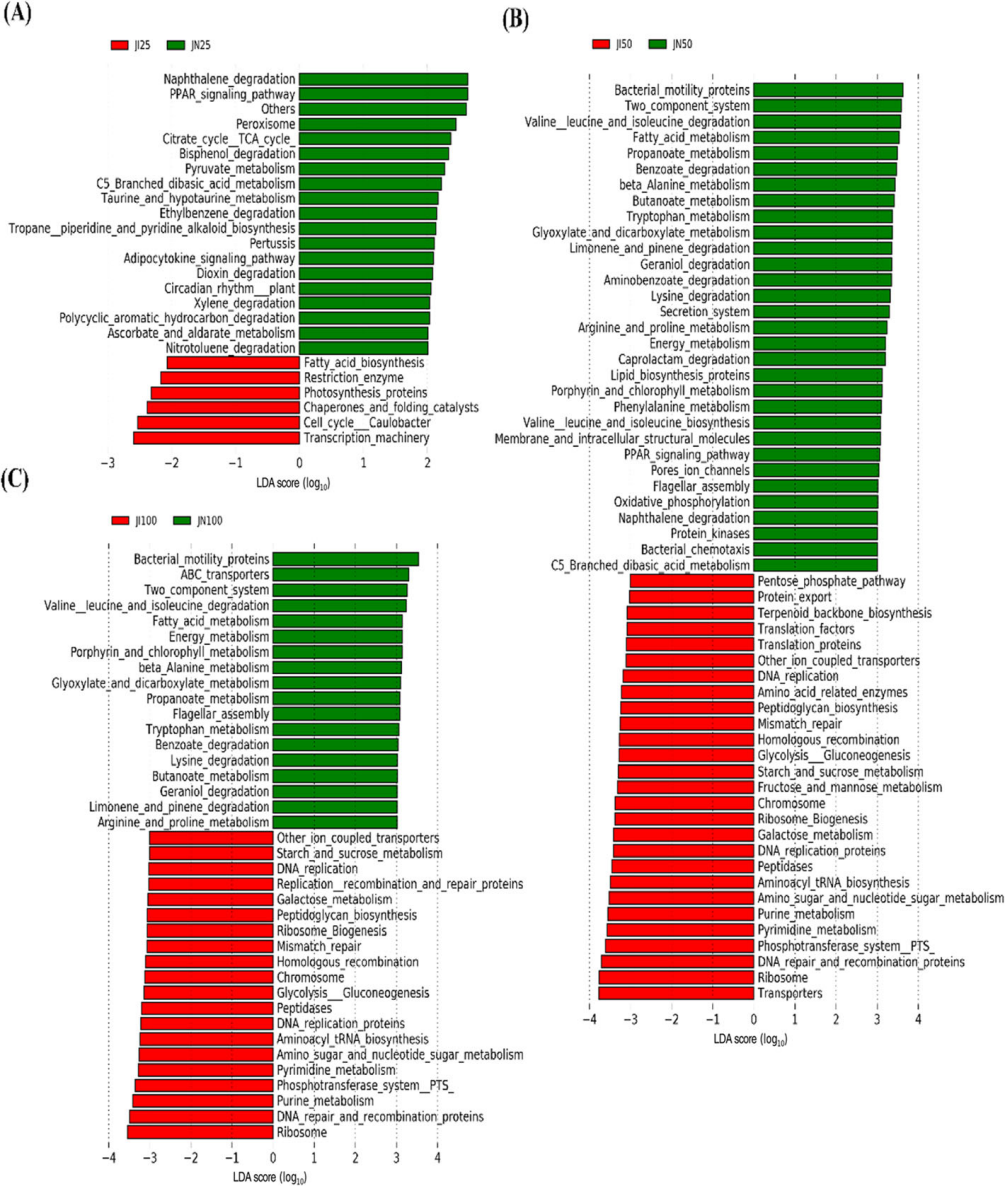
Predictive metagenomics showing differences in function between intrauterine growth retardation (IUGR) and normal birth weight (NBW) pigs in the jejunum using PICRUSt analysis (level 3). The data presented are obtained from 10 animals in each group (*n* = 10). JI and JN represent samples obtained from the jejunum of IUGR pigs and NBW pigs, respectively; 25, 50, and 100 represent 25, 50, and 100 kg BW groups. LEfSe analysis was utilized to identify potentially enriched pathways. Pathways with LDA scores ≥2 are shown in the 25 kg BW group (**a**), and pathways with LDA scores ≥3 in the 50 (**b**) and 100 (**c**) kg BW groups

PICRUSt analysis showed that metabolic pathways were classified into six functional categories (level 1) and used to compare the functional enrichment in IUGR pigs and NBW pigs (Fig. 7a). Moreover, we performed the same comparison by using 45 different gene functions (level 2) (Fig. 7b). Metabolism was the predominant functional pathway affected by the intestinal microbiota in both IUGR pigs and NBW pigs. However, the relative enrichment of different metabolic pathways in the intestinal microbiota appears to allow the discrimination between IUGR pigs and NBW pigs. LEfSe analysis was then performed to observe differences in gene functions (level 3; Fig. 8). Because many significantly enriched functional pathways were common between the 50 and 100 kg BW groups, we subsequently focused on these pathways in the jejunum and ileum of IUGR and NBW pigs.

In the jejunum, the expression of genes in the following categories was enriched in IUGR pigs compared to that in NBW pigs in the 50 and 100 kg BW groups (LDA > 3.0, *P* < 0.05): ion-coupled transporters, starch and sucrose metabolism, DNA replication, peptidoglycan biosynthesis, mismatch repair, homologous recombination, chromosome, galactose metabolism, phosphotransferase system, glycolysis/gluconeogenesis, purine metabolism, peptidases, DNA replication proteins, aminoacyl-t RNA biosynthesis, amino sugar and nucleotide sugar metabolism, pyrimidine metabolism, DNA repair and recombination proteins, ribosome function, and ribosome biogenesis. Conversely, the expression of genes in the following categories was enriched in NBW pigs compared to that in IUGR pigs in the 50 and 100 kg BW groups (LDA > 3.0, *P* < 0.05): bacterial motility proteins, two-component systems, degradation or metabolism of various amino acids, fatty acid metabolism, porphyrin and chlorophyll metabolism, beta-alanine metabolism, glyoxylate and dicarboxylate metabolism, propanoate metabolism, flagellar assembly, benzoate degradation, butanoate metabolism, geraniol degradation, and limonene and pinene degradation (Fig. 8).

**Fig. 9:**
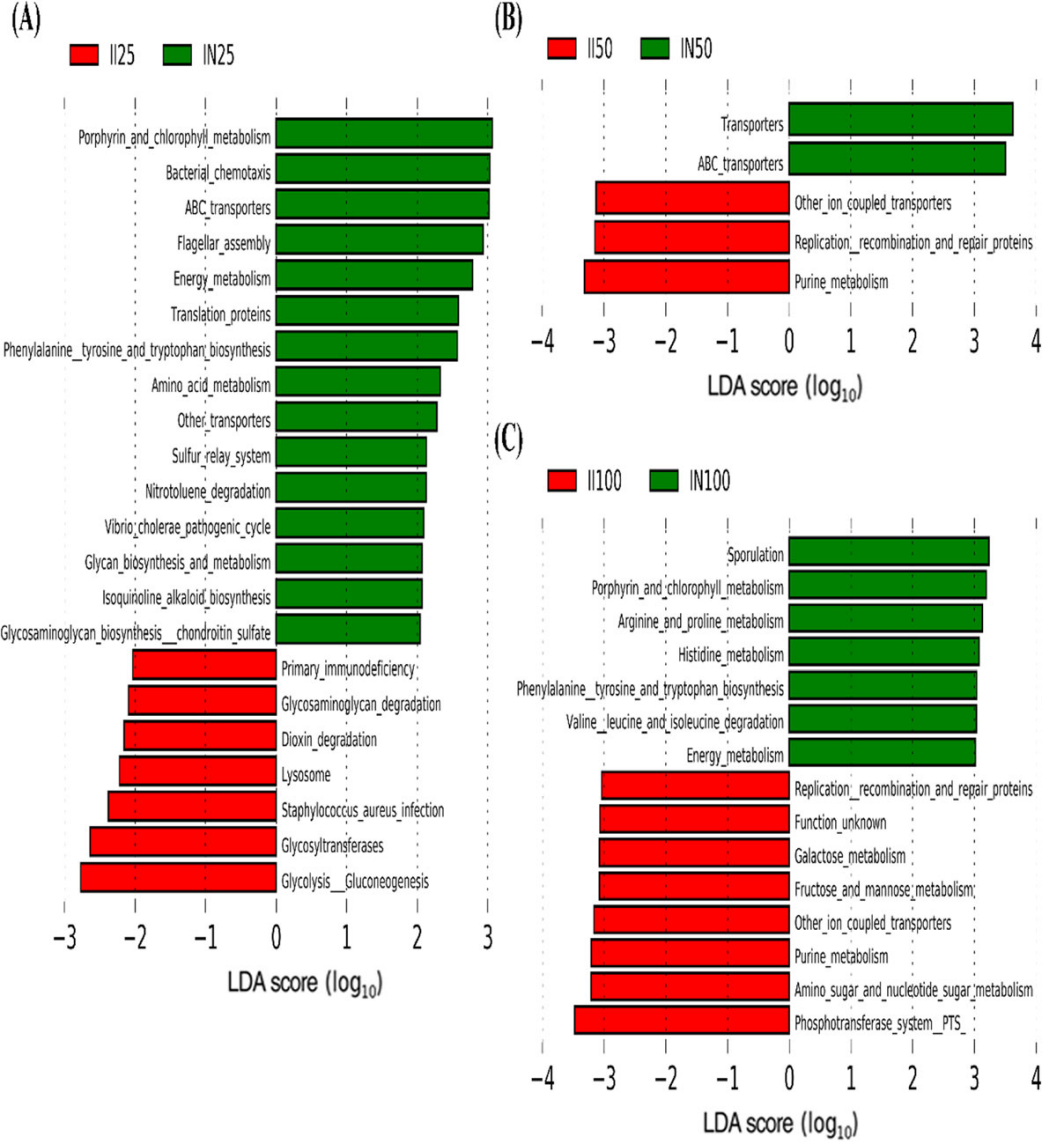
Predictive metagenomics showing differences in function between intrauterine growth retardation (IUGR) pigs and normal birth weight (NBW) pigs in the ileum using PICRUSt analysis (level 3). The data presented are obtained from 10 animals in each group (*n* = 10). II and IN represent samples obtained from the ileum of IUGR pigs and NBW pigs, respectively. Twenty-five, 50, and 100 represent 25, 50, and 100 kg BW. LEfSe analysis was used to identify potentially enriched pathways. Pathways with LDA scores ≥2 in the 25 kg BW group (**a**), and LDA scores ≥3 in the 50 (**b**) and 100 (**c**) kg BW groups are shown

In the ileum, the expression of genes involved in ion-coupled transporters, purine metabolism, and replication, recombination, and repair proteins was enriched in IUGR pigs compared to that in NBW pigs in the 50 and 100 kg BW groups (LDA > 3.0, *P* < 0.05). The ABC transporter pathway was enriched in NBW pigs compared to that in IUGR pigs in the 25 and 50 kg BW groups, and phenylalanine, tyrosine, and tryptophan biosynthesis and energy metabolism pathways were enriched in NBW pigs compared to that in IUGR pigs in the 25 and 100 kg BW groups (Fig. 9).

## Discussion

In a previous study, we reported alterations in the microbial communities of the small intestine in IUGR piglets at 7, 21, and 28 d of age, and that Bacteroidetes, *Bacteroides*, *Escherichia–Shigella*, and *Pasteurella* could be related to nutrient digestion and absorption, and growth and development regulation [14]. Microbial communities in the intestine are influenced by various environmental factors, including age, diet, lifestyle, and medication [17]. However, whether intestinal microbiota colonization during lactation has an effect during the growing-finishing period in IUGR pigs remains unclear. Thus, in the present study, we further analyzed the effects of IUGR on the microbial communities in the small intestine and plasma hormone profiles in growing-finishing pigs in the 25, 50, and 100 kg BW groups. Our study clearly showed that IUGR is associated with impaired growth after weaning, which is concomitant with alterations in the hormonal profile, intestinal microbiota alpha diversity, and microbiota composition.

Growth performance is usually greater when pigs are penned individually than penned in groups [18]. The reported factors related to reductions in ADFI and ADG in group-penned pigs include competition and aggressive behavior to maintain dominance hierarchy, an increase standing, and physiological responses due to chronic stress of weaning, competition, and aggressive encounters [19]. To reduce the influence of these factors, the pigs were individually penned in the present study. Specifically, IUGR piglets had significantly lower initial BW, weaned BW, final BW, ADG, and ADFI compared to NBW pigs in the 25, 50, and 100 kg BW groups, consistent with previous results [20, 21]. The decrease in ADG might have resulted from the reduced abundance of unclassified Peptostreptococcaceae, unclassified Clostridiales, unclassified Clostridiaceae, unclassified Pseudomonadaceae, and *Pseudomonas*, which are positively correlated with ADG. These results confirmed the known effects of IUGR on pig growth, as low birth weight leads to decreased postnatal growth rate.

Insulin promotes the synthesis of glycogen, fat, and protein [22]. The lower plasma insulin levels observed in IUGR pigs in the 25 kg BW group were consistent with those found in previous studies [23]. IUGR impairs both the exocrine and endocrine pancreatic development and reduces pancreatic weight in pigs [24], thereby concomitantly reducing plasma insulin levels [25]. However, the increase in plasma insulin in the 50 kg BW group may suggest that pancreatic impairment was restored during growth, in a manner similar to the observed increase in feed efficiency, and maybe also as the result of an adaptive process. Ghrelin is a GH-stimulating hormone, produced and secreted primarily from the stomach [26]. PP is a polypeptide hormone released by the pancreas, which suppresses appetite and food intake [27]. We found that IUGR pigs had increased plasma ghrelin and PP levels compared to NBW pigs in the 25 kg BW group, but these levels decreased in the 50 kg BW group, indicating that the kinetics of hormone secretion in IUGR pigs during growth is complex. A possible rationale for this result may be that the plasma hormone profiles may represent the early situation before plasma sampling, and that individual factors in IUGR and NBW pigs may lead to alterations in such profiles.

Intestinal microbiota alpha diversity has been associated with host health and metabolic capacity [28]. In pigs, intestinal bacteria diversity changes from weaning to growing and finishing stages [29]. We observed that alpha diversity of the jejunum and ileum microbiota in IUGR pigs was significantly higher than in NBW pigs in all BW groups evaluated, suggesting that IUGR pigs had a more diversified intestinal microbiota. A recent study showed that IUGR pigs had significantly higher alpha diversity (ACE and Chao1 indices) in the ileum than NBW pigs at 70 d of age [30]. The abundance of adherent bacteria in the intestinal mucosa of IUGR piglets has been shown to be higher than that in NBW piglets [31]. Accordingly, in the present study, the beta-diversity analysis showed that IUGR significantly altered the microbial community in the small intestine.

Firmicutes and Proteobacteria were the two most abundant microbial phyla detected in the ileum of both NBW pigs and IUGR pigs, in agreement with a previous study [32]. Firmicutes is associated with energy absorption from nutrition [33], while members of the Bacteroidetes phylum are specialized in the degradation of proteins and carbohydrates [34]. In the present study, the higher relative abundance of Firmicutes in IUGR pigs in the 50 and 100 kg BW groups, which was consistent with the results of a previous study [35], suggested that the intestinal microbiota of IUGR pigs may be more efficient than NBW pigs at recovering energy from the diet, notably for covering the high energy need of intestinal epithelium cell, although this needs to be validated experimentally. The Proteobacteria phylum includes a wide variety of pathogens (such as *Escherichia*, *Salmonella*, and *Yersinia*), and members of this phylum serve as indicators of intestinal inflammation and epithelial dysfunction [36]. Although IUGR pigs in the 50 and 100 kg BW groups showed a lower proportion of Proteobacteria in the jejunum compared to NBW pigs, their growth performance remained restricted. This phenomenon may be associated with impaired intestinal morphology and gastrointestinal dysfunction in IUGR pigs [8].

Within the Firmicutes phylum, *Lactobacillus* has been identified as a key genus in the intestine of pigs [37], with proportions ranging from 0.53–4.51% of the total ileal bacteria and 4.98–17.84% of the colonic bacteria in finishing pigs [38]. *Lactobacillus* utilizes fermentative carbohydrates and produces lactic acid as a major end product, thereby supplying energy to the cells of the intestinal epithelium [39]. In our study, IUGR pigs in the 50 and 100 kg BW groups had a significantly higher abundance of *Lactobacillus* in the small intestine than NBW pigs, but *Lactobacillus* abundance in the jejunum and ileum was negatively correlated with the growth performance. *Lactobacillus* is taxonomically complex and composed of over 170 species, 17 subspecies of which are occasional opportunistic pathogens [40]. For instance, *L. rhamnosus* and *L. rhamnosus GG* may be at the origin of *Lactobacillus* bacteremia [41]. These opposite roles of *Lactobacillus* species make it difficult to define clearly classify them as probiotics or pathogens for IUGR pigs. As a producer of short-chain fatty acids (SCFAs), the Ruminococcaceae family is responsible for the degradation of diverse polysaccharides and fibers. The production of SCFAs is considered beneficial for the maintenance of intestinal health [42]. Our findings showed that unclassified genera belonging to the Ruminococcaceae were the most abundant taxa in both IUGR pigs and NBW pigs. In the ileum of IUGR pigs in the 25 kg BW group, Ruminococcaceae showed higher abundance than in the ileum of NBW pigs during the growing period, in accordance with a recent study [30]. Dietary carbohydrates could be efficiently fermented by Ruminococcaceae during the growing stage, thereby enabling the bioavailability of luminal energy substrates. However, in a recent study, the mRNA expression of SCFA transporters (*FFAR1*, *FFAR2*, and *PPAR*) and their absorption capacity were decreased in IUGR pigs during the growth period [30]. Thus, the higher amounts of SCFAs fermented by Ruminococcaceae might not be totally absorbed and utilized by the intestinal epithelial cells effectively, which leads to nutrient loss in IUGR pigs during the growing period. *Ochrobactrum* is a genus belonging to the Proteobacteria phylum containing 15 species [43], of which *O. tritic* and *O. intermedium* are emerging pathogens capable of causing infections in healthy individuals [44]. We found that IUGR pigs in the 25 and 50 kg BW groups had a lower abundance of *Ochrobactrum* in the jejunum than NBW pigs. However, if such a low abundance of *Ochrobactrum* in IUGR piglets confers a beneficial effect with respect to infection remains to be determined.

In the present study, the abundance of several bacteria, such as Firmicutes, Ruminococcaceae, and *Lactobacillus*, was increased in the jejunum and ileum, which may lead to an increased production of SCFAs and lactic acid to supply extra energy to the intestinal epithelial cells of IUGR pigs. This phenomenon could be explained with the “thrifty phenotype” hypothesis, which suggests that when nutritional conditions in the uterus are suboptimal, the growth and metabolism of the fetus are restricted. However, when the postnatal nutritional condition is adequate, IUGR pigs undergo a catch-up growth with respect to the intestinal microbiota [45]. This phenotype is presumably the result of an adaptation to adverse conditions. However, the energy supply through microbial fermentation from luminal substrates remains an inefficient method of digesting nutrients and may lead to an imbalanced nutrient supply in IUGR pigs [30]. Due to the multifaceted effect of impaired organ development, intestinal morphology, insufficient maternal colostrum intake, and *Lactobacillus* bacteremia, among others [46], the energy from microbial fermentation is insufficient to prevent growth delay, thereby leading to restricted growth performance during the growing-finishing period of IUGR pigs. Despite the growth compensation by beneficial intestinal microbiota colonization in IUGR pigs when a high quality diet was provided, a lower return in production performance over investment value was still observed for these pigs.

In the present study, PICRUSt analysis showed that IUGR altered several microbial gene functions. Carbohydrate and amino acid metabolism were the dominant functions in both IUGR pigs and NBW pigs, likely because the main ingredients of the feed and substrate type of predominant microbial fermentation are carbohydrates and proteins. Interestingly, intestinal microbiota of IUGR pigs displayed, in terms of gene expression, a presumably higher capacity for carbohydrate metabolism, translation, nucleotide metabolism, galactose metabolism, glycan biosynthesis and metabolism, DNA replication and repair, and cellular processes and signaling. The higher expression in IUGR pigs of genes involved in carbohydrate metabolism and glycan biosynthesis and metabolism was associated with a higher proportion of Firmicutes and *Lactobacillus*. *Lactobacillus* use fermentative carbohydrates to produce lactic acid [39], while Firmicutes can metabolize proteins and carbohydrates to produce SCFAs [35]. Moreover, we found that the expression of genes involved in DNA replication, mismatch repair, chromosome and homologous recombination, and ribosome biogenesis was upregulated in the jejunum microbiota of IUGR pigs in the 50 and 100 kg BW groups, suggesting that IUGR might regulate gene expression in the intestinal bacteria, in agreement with the results of a previous study [32].

Interestingly, when compared to NBW pigs, IUGR pigs showed lower expression of genes involved in lipid metabolism, glyoxylate and dicarboxylate metabolism, amino acid metabolism, energy metabolism, terpenoid and polyketide metabolism, xenobiotic biodegradation and metabolism, cofactor metabolism, and vitamin metabolism, consistent with the results of our previous study [14]. The downregulation of amino acid metabolism is consistent with lower concentrations of most amino acids observed in IUGR pigs [47]. Because of this, dietary supplementation with functional amino acids (e.g. arginine, tryptophan, and lysine) may help to mitigate growth restriction and improve the health and growth of IUGR pigs [48, 49].

Further studies are needed to determine the possible causal links between the changes observed in the intestinal microbiota and the changes in the measured physiological parameters. Consistent with this hypothesis, different bacterial metabolites have been shown to modify the secretion of various hormones produced by the enteroendocrine cells of the intestinal epithelium [50]. Moreover, lower feed intake may have an impact on nutrient availability, due to the metabolic activity of the intestinal microbiota [51].

## Conclusion

In this study, IUGR pigs presented a lower growth performance, higher microbial alpha diversity, and different plasma concentrations of hormones during the growing-finishing period, as compared to NBW pigs. Alterations in the abundances of Firmicutes, Proteobacteria, Ruminococcaceae, *Lactobacillus,* and *Ochrobactrum* in the small intestine may be associated with IUGR, and could therefore serve as a future target for intestinal microbiota interventions in growing-finishing pigs subjected to IUGR.

## Supplementary information

**Additional file 1: Figure S1.** Differences in microbial community structures in the jejunum (A) and ileum (B) between intrauterine growth retardation (IUGR) pigs and normal birth weight (NBW) pigs throughout the trial. The data presented are obtained from 10 animals in each group (*n*=10). Rarefaction curve analysis was used to evaluate whether further sequencing would likely detect additional taxa. JI and JN represent samples obtained from the jejunum of IUGR pigs and NBW pigs, respectively; II and IN represent samples obtained from the ileum of IUGR pigs and NBW pigs, respectively. 25, 50, and 100 represent 25, 50, and 100 kg BW groups.

## Data Availability

The data generated or analyzed during the current study are available from the corresponding author by reasonable request.

## Abbreviations

BW: Body weight
IUGR: Intrauterine growth restriction
LEfSe: Linear discriminant analysis effect size
NBW: Normal birth weight
OTU: Operational taxonomic units
SCFA: Short-chain fatty acid
