# Supplementary material for: Intrauterine growth restriction alters growth performance, plasma hormones, and small intestinal microbial communities in growing-finishing pigs
Source: J Anim Sci Biotechnol. 2020 Aug 19;11:86. doi: 10.1186/s40104-020-00490-x (PMC7437023; doi:10.1186/s40104-020-00490-x)
Supplement: Supplementary file 1 — Additional file 1: Figure S1. Differences in microbial community structures in the jejunum (A) and ileum (B) between intrauterine growth retardation (IUGR) pigs and normal birth weight (NBW) pigs throughout the trial. The data presented are obtained from 10 animals in each group (n=10). Rarefaction curve analysis was used to evaluate whether further sequencing would likely detect additional taxa. JI and JN represent samples obtained from the jejunum of IUGR pigs and NBW pigs, respectively; II and IN represent samples obtained from the ileum of IUGR pigs and NBW pigs, respectively. 25, 50, and 100 represent 25, 50, and 100 kg BW groups. [file 40104_2020_490_MOESM1_ESM.docx]

**Intrauterine growth restriction alters growth performance, plasma hormones, and small intestinal microbiota communities in growing-finishing pigs**

Liang Xiong^1,2^, Jinming You^2^, Wanghong Zhang^1^, Qian Zhu^1^, Francois Blachier^3^, Yulong Yin^1^, Xiangfeng Kong^1^^[[1]](#footnote-1)^

^1^ CAS Key Laboratory of Agro-ecological Processes in Subtropical Regions, Hunan Provincial Key Laboratory of Animal Nutritional Physiology and Metabolic Process, National Engineering Laboratory for Pollution Control and Waste Utilization in Livestock and Poultry Production, Institute of Subtropical Agriculture, Chinese Academy of Sciences, Changsha, Hunan 410125, China

^2^ Key Laboratory of Animal Nutrition in Jiangxi Province, College of Animal Science and Technology, Jiangxi Agricultural University, Nanchang, Jiangxi 440000, China

^3^ Université Paris-Saclay, AgroParisTech, INRAE, UMR PNCA, 75005, Paris, France

**
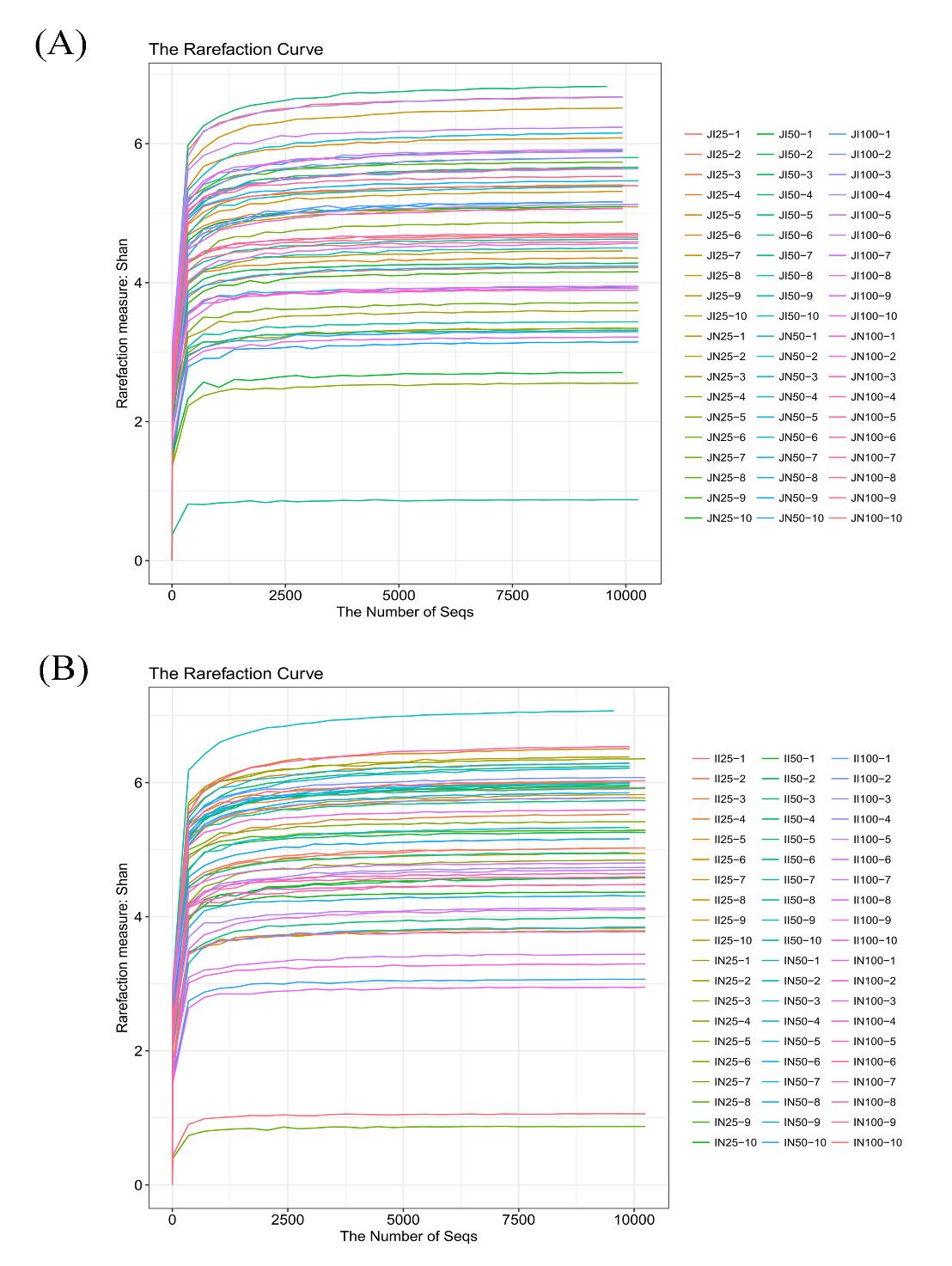
**

**Supplementary Figure 1** Differences in microbial community structures in the jejunum (A) and ileum (B) between intrauterine growth retardation (IUGR) pigs and normal birth weight (NBW) pigs throughout the trial. The data presented are obtained from 10 animals in each group (n=10). Rarefaction curve analysis was used to evaluate whether further sequencing would likely detect additional taxa. JI and JN represent samples obtained from the jejunum of IUGR pigs and NBW pigs, respectively; II and IN represent samples obtained from the ileum of IUGR pigs and NBW pigs, respectively. 25, 50, and 100 represent 25, 50, and 100 kg BW groups.

1. Corresponding author: Professor Xiangfeng Kong, E-mail: nnkxf@isa.ac.cn [↑](#footnote-ref-1)
